# Antioxidant potential and optimization of production of extracellular polysaccharide by *Acinetobacter indicus* M6

**DOI:** 10.1186/s43141-021-00137-y

**Published:** 2021-03-12

**Authors:** Ch. Ravi Teja, Abraham P. Karlapudi, Neeraja Vallur, K. Mamatha, D. John Babu, T. C. Venkateswarulu, Vidya Prabhakar Kodali

**Affiliations:** 1 Department of Biotechnology, Vikrama Simhapuri University, Kakutur, Nellore, A.P-524320 India; 2 Department of Biotechnology, VFSTR University, Vadlamudi, Guntur, A.P-522213 India; 3 SRR and CVR Government Degree College, Machavaram, Vijayawada, A.P-520010 India

**Keywords:** Extracellular polysaccharide, Antioxidant activity, Response surface methodology, Monosaccharide composition

## Abstract

**Background:**

Extracellular polysaccharides (ECPs) produced by biofilm-producing marine bacterium have great applications in biotechnology, pharmaceutical, food engineering, bioremediation, and bio-hydrometallurgy industries. The ECP-producing strain was identified as *Acinetobacter indicus* M6 species by 16S rDNA analysis. The polymer produced by the isolate was quantified and purified and chemically analyzed, and antioxidant activities have been studied. The face-centered central composite design (FCCCD) was used to design the model.

**Results:**

The results have clearly shown that the ECP was found to be endowed with significant antioxidative activities. The ECP showed 59% of hydroxyl radical scavenging activity at a concentration of 500 μg/mL, superoxide radical scavenging activity (72.4%) at a concentration of 300 μg/mL, and DPPH**˙** radical scavenging activity (72.2%) at a concentration of 500 μg/mL, respectively. Further, HPLC and GC-MS results showed that the isolated ECP was a heteropolymer composed of glucose as a major monomer, and mannose and glucosamine were minor monomers. Furthermore, the production of ECP by *Acinetobacter indicus* M6 was increased through optimization of nutritional variables, namely, glucose, yeast extract, and MgSO_4_ by “Response Surface Methodology”. Moreover the production of ECP reached to 2.21 g/L after the optimization of nutritional variables. The designed model is statistically significant and is indicated by the *R*^2^ value of 0.99. The optimized medium improved the production of ECP and is two folds higher in comparison with the basal medium.

**Conclusions:**

*Acinetobacter indicus* M6 bacterium produces a novel and unique extracellular heteropolysaccharide with highly efficient antioxidant activity. GC-MS analyses elucidated the presence of quite uncommon (1→4)-linked glucose, (1→4)-linked mannose, and (→4)-GlcN-(1→) glycosidic linkages in the backbone. The optimized medium improved the production of ECP and is two folds higher in comparison with the basal medium. The newly optimized medium could be used as a promising alternative for the overproduction of ECP.

## Background

*Acinetobacter* species produce medicinally and commercially important diverse group of molecules [1]. Extracellular polysaccharides (ECPs) are one of such important molecules. ECPs are long-chain, high molecular-mass polymers which have been reported to show antiulcer, immunomodulatory, antiviral, antioxidant, and various other biological activities [2, 3]. These ECPs are the alternative class of biothickeners and also proved to have good emulsifying property apart from the texture-promoting property in various foods [4]. In some countries of the European Union (EU) and the United States of America (USA), where addition of synthetic texture-promoting agents in food and dairy products is prohibited, ECPs can successfully be used as food additives to enhance texture [5]. The ECPs are economically important because they can impart functional effects to foods. Depending on the monosaccharide composition, ECPs can be classified into homo (HoPSs) and heteropolysaccharides (HePSs). HoPSs consist of only one type of monosaccharide.

Recently, studies have been focused on antioxidant polysaccharides that can find potential applications in food industries [6]. Free radicals such as superoxide radical anion (O_2_^·−^), hydroxyl radical (OH·), and other reactive oxygen species (ROS) are considered to be highly potent oxidants that can react with all biomacromolecules in living cells, and they may associate with carcinogenesis and mutagenesis [7]. ECPs were reported to have free radical scavenging activities [8]. Bacterial polysaccharides isolated from *Pantoea agglomerans* and *Microbacterium terregens* [9] showed pronounced antioxidant activities.

Considering the antioxidant activity and other therapeutic importance of the ECP, it is important to characterize the ECP structurally. Therefore, the monomeric composition of the ECP was identified by HPLC, and glycosidic linkages of the ECP were determined by GC-MS. HPLC is frequently used for both the qualitative and quantitative analysis of liberated monosaccharides after acid hydrolysis. HPLC with an ultraviolet (UV) detector or refractive index (RI) is an alternative method for quantitative determination of saccharides. Due to low sensitivity and inapplicability to gradient elution, HPLC with RI detector is less commonly used [10]. The alternative approach is HPLC with UV detector, which is highly sensitive and widely used for analysis and quantification of monomers. GC attached to a mass spectrometer is an efficient and widely applicable method for linkage analysis of methylated polysaccharides. Methylation analysis is an essential step for studying the linkage pattern of sugar residues. Depending on the mass spectra obtained, the glycosidic linkages are analyzed [11, 12].

The production of polymers is highly influenced by different factors such as nutritional variables and physical variables of the process, namely, temperature, pH, RPM, dissolved oxygen concentration, and RPM [9]. Studies also reported that the growth and development of film formation depend on surface area, smoothness, flow velocity, and nutrients [13]. Very few reports are available on design fermentation medium through response surface methodology (RSM) optimization studies for production of ECPs [13]. Therefore, the present study aimed to design the low-cost fermentation medium for enhanced production of the ECP using RSM.

Considering the tremendous reported therapeutic and commercial potentials, the ECP molecule may be developed as a potential drug molecule in the near future. Establishing the structure-function relationship of the ECP molecule by elucidating its complete molecular structure on further chemical derivatizations and enzymatic digestions (with glucosidases) will enable to identify the right fragments of the large ECP molecule, responsible for important bioactive properties of therapeutic and commercial interests. This would also help to use the small fragments for specific therapeutic purposes, instead of using the whole molecule.

## Methods

### Extraction, purification, and quantification of extracellular polysaccharide

ECP-producing organism was isolated and identified as mentioned previously [14]. Ten milliliters of overnight culture was centrifuged, and 30 mL alcohol (95%) was added to the supernatant. The mixture was shaken thoroughly and kept at 4 °C for overnight. The precipitated polymer was separated by centrifugation and dried to get crude ECP. The dried and crude powder (10 mg) was dissolved in 1 mL 0.2 M NaCl buffer to a concentration of 10 g/L and was filtered through a 0.22-μm membrane filter, loaded onto a Sephadex G-100 column (Sigma Aldrich, St Louis, USA-50 × 1.5 cm). The column was eluted with the same buffer at a flow rate of 0.5 mL/min, and 0.5 mL of fractions was collected. Total carbohydrate content of the fractions was determined by phenol-sulfuric acid method, and the carbohydrate content was measured by phenol sulfuric acid method [15].

### Antioxidant activity of the ECP

The antioxidant potential of ECP was studied by superoxide radical scavenging assay by phenazine methosulfate (PMS)-nicotinamide adenine dinucleotide (NADH)-Nitroblue tetrazolium chloride (NBT) system, 1,1-diphenyl-2-picrylhydrazyl (DPPH) radical scavenging activity [16]. Vitamin C was used as a positive control.

### Hydroxyl radical scavenging activity of the ECP

The hydroxyl radical scavenging activity of ECP was measured according to Liu et al. and Ren et al. [17, 18]. The hydroxyl radicals were generated in the l-ascorbic acid-CuSO_4_ system by reduction of Cu^2+^ and were assayed by the oxidation of cytochrome C. In this experiment, hydroxyl radicals were generated in 3 mL of 0.15 mM sodium phosphate buffer (pH 7.4), which included 100 μM L-ascorbic acid, 100 μM CuSO_4,_ 12 μM cytochrome C, and the samples to be tested at different concentrations. The mixture was incubated at 25 °C for 90 min. The color change of cytochrome C was measured at 550 nm. Thiourea was used as control, and glucose was used as negative control.

The inhibition rate of hydroxyl radical generation by thiourea was taken as 100%.

The inhibition rate was calculated using the following equation:

$$ \mathrm{Inhibition}\ \mathrm{rate}\ \left(\%\right)=\left[\frac{T-{T}_2}{T-{T}_1}\right]\mathrm{x}100 $$

where *T* is the transmittance of hydroxyl radical (OH^**·**^) generation system and *T*_1_ and *T*_2_ are the transmittance of control and test sample systems respectively.

### Superoxide (O_2_^·^) radical scavenging activity of the ECP

Measurement of superoxide radical scavenging activity was done based on the method described by Ren et al. [18] and Nishimiki et al. [19]. To 1 mL of NBT solution (156 μM NBT in 100 mM phosphate buffer, pH 7.4), 1 mL NADH solution (468 μM in 100 mM phosphate buffer, pH 7.4) and 0.1 ml of ECP in water were added and mixed well. The reaction was started by adding 100 μL of PMS solution (60 μM PMS in 100 mM phosphate buffer, pH 7.4) to the mixture. The reaction mixture was incubated at 25 °C for 5 min, and the absorbance was measured at 560 nm against blank samples using a spectrophotometer, and vitamin C was used as positive control. Decreased absorbance of the reaction mixture indicated increased superoxide anion-scavenging activity.

The scavenging activity of superoxide radical (%) was calculated from the following equation:

$$ \mathrm{Superoxide}\ \mathrm{radical}\ \mathrm{scavenging}\ \mathrm{activity}\ \left(\%\right)=\left[1-\frac{A_{Sample}}{A_{Blank}}\right]\mathrm{x}100 $$

where *A*_Blank_ is the absorbance of the control reaction (containing all reagents except the test compound) and *A*_Sample_ is the absorbance of the test compound.

### DPPH radical scavenging activity

The free radical scavenging activity of ECP was measured by DPPH free radical scavenging assay. To 1 mL of 0.1 mM solution of DPPH in ethanol, 3 ml of ECP in water was added in different concentrations. After 30 min, absorbance was measured at 517 nm against blank. Radical scavenging activity was expressed as percentage inhibition of DPPH and estimated by the following equation [20]:

$$ \mathrm{DPPH}\ \mathrm{free}\ \mathrm{radical}\ \mathrm{scavenging}\ \mathrm{activity}\ \left(\%\right)=\left[1-\frac{A_{Sample}}{A_{Blank}}\right]\mathrm{x}100 $$

where *A*_Blank_ is the absorbance of the control reaction (containing all reagents except the test compound) and *A*_Sample_ is the absorbance of the test compound. Vitamin C was used as the positive control. All determinations were performed in triplicate. Decrease in the absorbance indicated the antioxidant activity.

### Compositional analysis of ECP

HPLC analysis is a commonly used method to determine the monosaccharide composition of polysaccharides. The polysaccharides are hydrolyzed to get individual monomers and then labeled with anthranilic acid to increase the florescence for easy and accurate identification [21]. The sugar composition of the ECP was studied by high-performance liquid chromatography (HPLC) [7]. For specific determination of monosaccharides with high sensitivity, ECP was acid hydrolyzed and then derivatized in a simple step with excess anthranilic acid (2-aminobenzoic acid) in the presence of sodium cyanoborohydride to give highly fluorescent-stable derivatives. The monosaccharide derivatives were completely separated from the excess reagent and from each other by HPLC on a C_18_ reversed-phase column using a butylamine-phosphoric acid-tetrahydrofuran mobile phase [2].

### Acid hydrolysis of ECP

ECP isolated from 36 h culture was dissolved in deionized water and dialyzed against deionized water using 12 kDa membrane at 4 °C for 24 h and then lyophilized. Ten milligrams of lyophilized ECP was hydrolyzed with 2 M trifluoroacetic acid (TFA) for 6 h at 100 °C. TFA was removed using rotary vacuum evaporator [22].

### Derivatization of monosaccharides with anthranilic acid

The hydrolysates were derivatized by anthranilic acid reagent [21, 22]. Briefly, anthranilic acid reagent was prepared by dissolving anthranilic acid (30 mg) and sodium cyanoborohydride in 1 mL of acetate-boric acid-methanol solution. The ECP hydrolysates were dissolved in 100 μL of 1% sodium acetate, and an aliquot of 50 μL was transferred to a new screw-cap freeze vial. Samples were mixed with 100 μL of anthranilic acid and capped tightly. Vials were heated at 80 °C for 60 min. After cooling, the samples were made up to 1 mL with solvent A (2% 1-butylamine, 0.5% phosphoric acid, and 1% tetrahydrofuran) for analysis in HPLC.

### HPLC analysis of anthranilic acid-monosaccharide derivatives

Monosaccharide derivatives were analyzed in Agilent 1000 series HPLC (Agilent Technologies, Model No.1100). A reversed-phase C_18_ column (ZORBAX 300 SB-C18, 5 μm, 4.6 × 250 mm, USA) and 1-butylamine-phosphoric acid-tetrahydrofuran mobile phase system consisting of solvents A and B were used for this analysis. Solvent A comprises of 0.2% 1-butylamine, 0.5% phosphoric acid, and 1% tetrahydrofuran in water, and solvent B consisted of equal parts of solvent A and acetonitrile. The separations were carried out at 24 °C using a flow rate of 1 mL/min, and 20 mL of each sample was injected. An UV detector was used to detect the derivatized monosaccharides. The gradient program was used according to [21].

### Determination of monosaccharide linkage analysis of the ECP molecule

#### GC-MS methylation analysis

A solution of ECP (5 mg) in dimethyl sulfoxide (0.5 mL) was permethylated by adding finely powdered NaOH (20 mg) and methyl iodide (0.1 mL). Then, the mixture was sonicated for 15 min. The permethylated ECP was extracted with CHCl_3_ (1 mL) and H_2_O (3 mL). CHCl_3_ phase was separated and dried under N_2_ and hydrolyzed in 2 M TFA at 100 °C for 1 h. The hydrolyzed ECP was reduced with 50 mM NaBH_4_ at room temperature for 4 h and evaporated three times from a mixture of acetic acid/methanol (1:1) followed by acetylation with 50:50 acetic anhydride/pyridine at 100 °C for 90 min. Alditol acetates of the methylated sugars were analyzed by Shimadzu GCMS-QP2010. The temperature program and other column conditions were used according to Kim et al. [23, 24].

### Optimization studies of ECP production

#### Production of ECP in shake flask

Luria Bertani medium was inoculated with *Acinetobacter indicus* M6, incubated for 48 h at 160 rpm at room temperature. Effect of salt, carbon, and nitrogen source on ECP production has been studied by varying concentrations of MgSO_4_, glucose, and yeast extract. ECP production was expressed in terms of total carbohydrate concentration spectrophotometrically (490 nm) [18]. ECP extraction method was followed as mentioned above.

### Optimization of nutritional variables by RSM

Response surface methodology (RSM) is a more convenient tool for designing experiments, plotting models, evaluating the effects of factors, and exploring optimum conditions of factors for significant responses. RSM is also used for optimization of prominent varieties of fermentation media and studying interactions among various bioprocess parameters with the minimum number of experiments [25, 26]. In the present experimentation, production of ECP in shake flask culture with *Acinetobacter indicus* M6 was found to be higher when compared to other bacterial species. Therefore, this strain has been considered as a potential bacterium for ECP production and hence it has been aimed to develop a suitable medium for enhanced production of ECP through optimization of nutrient component concentrations by response surface methodology (RSM). Fermentation medium variables such as glucose (A), yeast extract (B), and MgSO_4_ (C) were optimized using the Design Expert software (Version 7.0.0, Stat-Ease Inc., Minneapolis, USA). The variables range from low (−1) to high (+ 1) used in the study are presented in Table 1.The impact of nutritional components on production and regression analysis of experimental data was carried out; further, the three-dimensional surface plots were drawn. The model was validated through the conduction of an experiment at predicted variables as suggested by the designed statistical model [27].

**Table 1:** Carbohydrate and protein contents of crude and pure ECP

S.No	Fraction	Total carbohydrate (%)	Protein (%)
1.	Crude ECP^a^	92 ± 2	0.9 ± 0.3
2.	Purified ECP^b^	98 ± 1	0

^a^Alcohol-precipitated ECP

^b^Purified ECP by gel filtration chromatography

## Results

### Extraction, purification, and quantification of the ECP

**Fig. 1:**
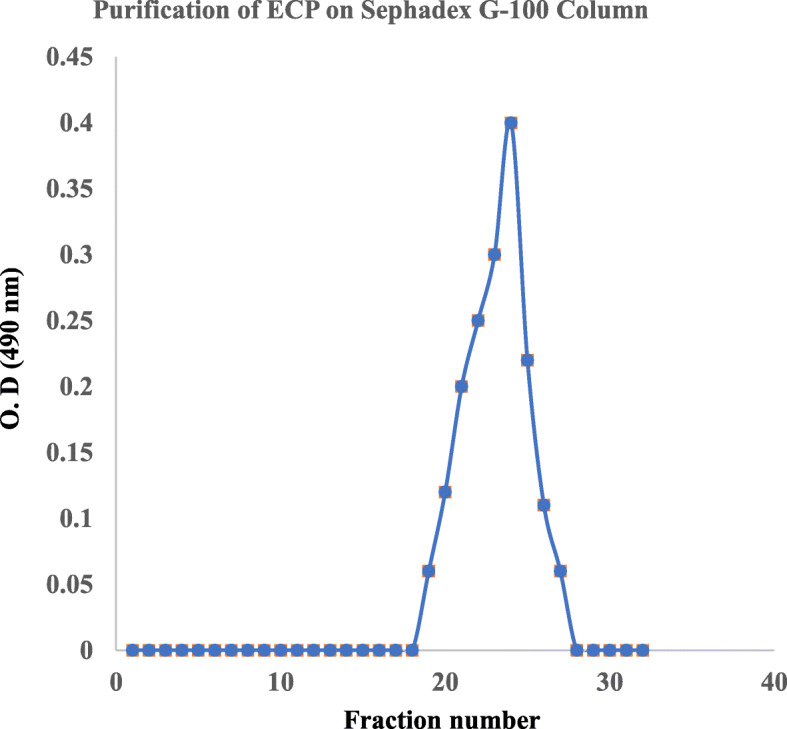
Purification of ECP on Sephadex G-100 Column. 10 mg/ml was loaded and 0.5 ml was collected

The ECP produced by *Acinetobacter indicus* M6 was extracted as described previously and then purified. The gel filtration chromatogram (Fig. 1) showed that the elution of the ECP starts at the 18th fraction and ended at the 28th fraction. There was no protein content in polysaccharide fractions, indicating that the ECP had no associated proteins. The carbohydrate and protein contents of the crude and purified ECP are listed in Table 1. While the total sugar content (%, w/w) of the ECP was found to increase by 6% on purification, the % content of the contaminating proteins decreased to zero from a value of 0.9%. The total carbohydrate and protein concentrations were observed to be 380 μg/mL and 150 μg/mL respectively.

### Antioxidant activity of the ECP

#### Hydroxyl radical scavenging assay of the ECP

**Fig. 2:**
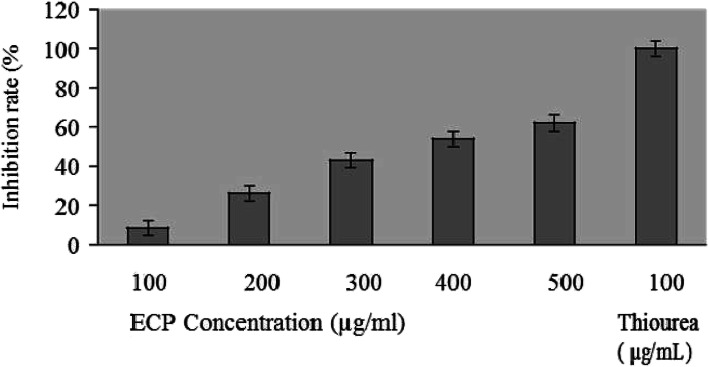
OH radical scavenging activity of the ECP. Values are means of triplicates ± SD. The rate of inhibition of hydroxyl radical generation by thiourea (100 μg/ml) was taken 100%

The hydroxyl radical scavenging activity of ECP (ranging from 100 to 500 μg/mL) by ascorbic acid-Cu^2+^-cytochrome C system is shown in Fig. 2. The scavenging activity of the ECP when correlated with its concentration showed that the activity increased till a concentration of 500 μg/mL and remained stable thereafter. ECP showed considerable hydroxyl radical scavenging activity of 59% at the concentration of 500 μg/mL. Here, in these experiments, the rate of inhibition of hydroxyl radical generation by thiourea (as a positive control) at a concentration of 100 μg/mL was taken as 100%, and glucose was used as negative control.

#### Superoxide (O_2_˙^−^) radical anion scavenging activity of the ECP

**Fig. 3:**
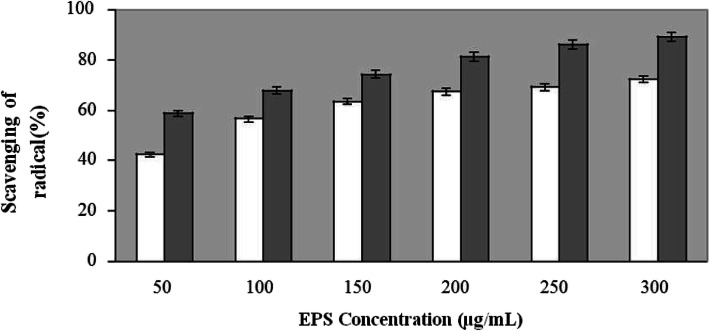
Scavenging activity of ECP against superoxide radical generated in PMS/NADH system with vitamin C used as positive control. Values are means of triplicates ± SD. White bar indicates ECP, Black bar indicates Vitamin C

The decrease in absorbance at 560 nm with the addition of ECP indicates the consumption of superoxide radical in the reaction mixture (Fig. 3). The ECP showed noticeable superoxide radical scavenging activity with increasing concentration ranging from 50 to 300 μg/mL. The maximum scavenging activity of the ECP was estimated to be 72.4% where reference compound vitamin C showed 90% at concentration of 300 μg/mL.

#### DPPH free radical scavenging activity

**Fig. 4:**
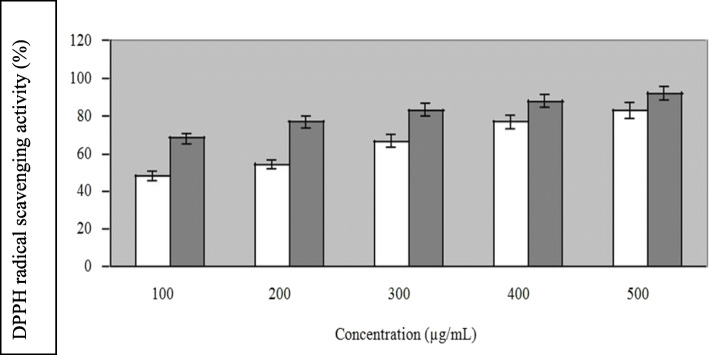
Scavenging effects of ECP against 1, 1-diphenyl-2-picryl hydrazyl radical with vitamin C used as positive control. Values are means of triplicates ± SD. White bar indicates ECP, Black bar indicates Vitamin C

This method is based on the reduction of methanolic DPPH^**·**^ solution in the presence of a hydrogen-donating antioxidant, due to the formation of the non-radical form DPPH-H by the reaction. DPPH^**·**^ is a stable free radical and accepts an electron or hydrogen radical to become a stable diamagnetic molecule [21, 22]. The antioxidant of ECP was determined by DPPH^**·**^ radical scavenging activity assay. Vitamin C was used as standard. Figure 4 illustrates the DPPH**˙** radical scavenging ability of ECP which increased with increasing concentration of ECP ranging from 100 to 500 μg/mL. Vitamin C and ECP showed 92% and 72.2% DPPH**˙** radical scavenging activity respectively at 500 μg/mL concentration. In terms of this antioxidant activity, the antioxidant activity of the ECP and the standard were comparable.

### Compositional analysis of the ECP

**Fig. 5:**
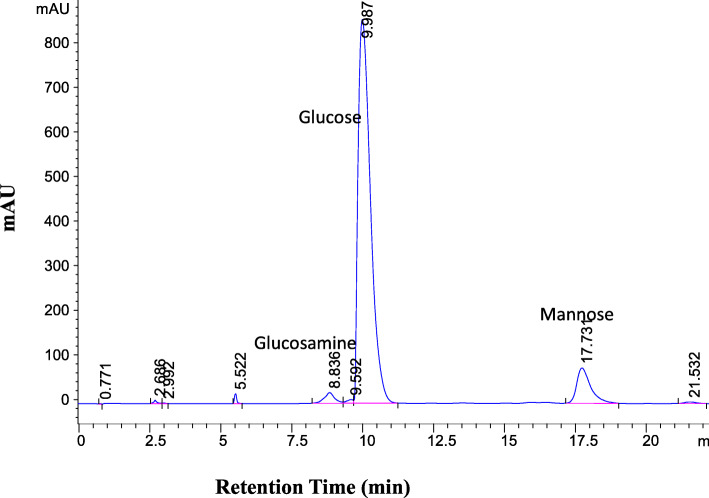
HPLC analysis of hydrolyzed ECP. (Glucose is a major monomeric unit; mannose and glucosamine are the minor units)

The ECP after being hydrolyzed and derivatized with anthranilic acid was analyzed for its sugar composition by HPLC. The HPLC chromatogram (Fig. 5) showed that the ECP was a heteropolysaccharide, composed of glucose and glucosamine [22]. In terms of peak area, glucose (retention time 9.9 min) was the major monosaccharide, whereas glucosamine (retention time 8.8 min), and mannose (retention time 17.7) were the minor ones.

### Determination of linkages between the monosaccharide units

**Fig. 6:**
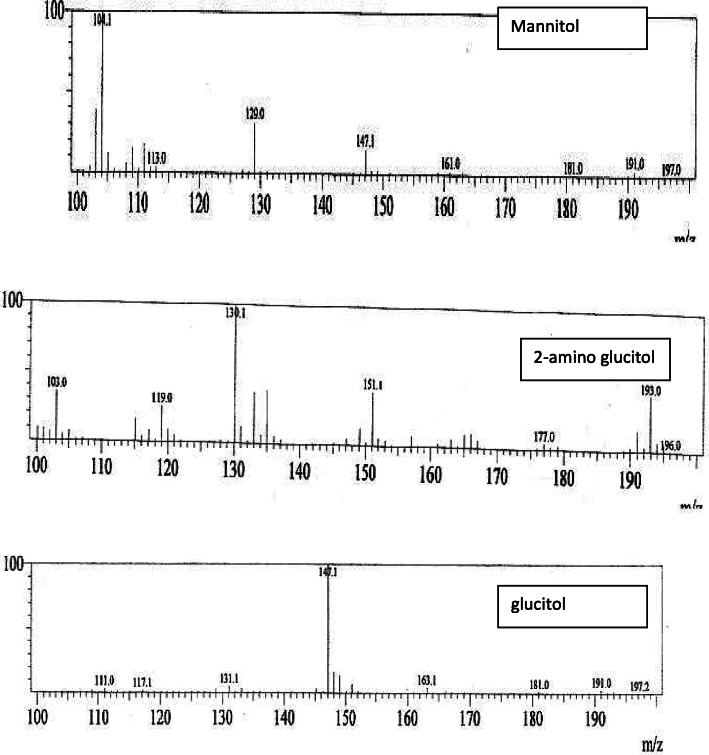
Mass spectra obtained by GC-MS analysis obtained by Shimadzu GC-MS QP 2010 using ZB-1 column

**Fig. 7:**
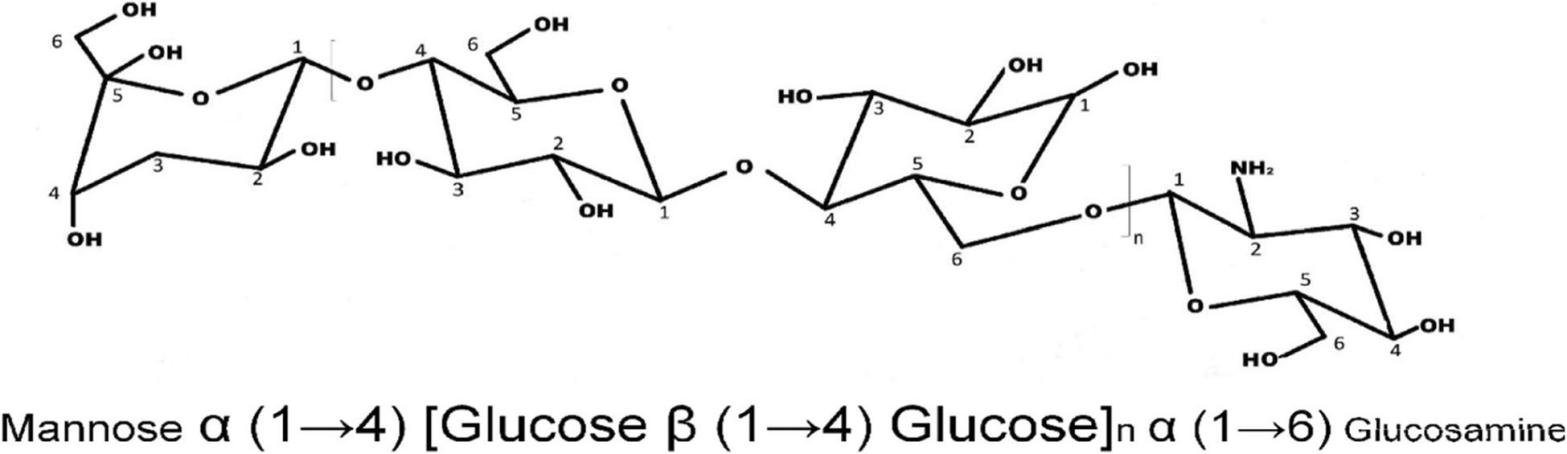
The probable structure of ECP

The sugar linkages in alditol acetates of the methylated sugars of the ECP were elucidated by GC-MS analysis. Methylation analysis is a widely used method for determining polysaccharide structure [28, 29]. However, reductive cleavage depolymerization has several advantages compared to standard methylation analysis, and it is an effective method for structural characterization of complex carbohydrates, which have different sugar residues [27]. The alditol derivatives were 1,2,4-tri-*O*-acetyl-3,6-tri-*O*-methyl-D-mannitol; 1,4-di-*O*-acetyl-2,4,6-tri-*O*-methyl-D-glucitol; 1,4,6-di-*O-*acetyl-3-mono-*O*-methyl-2-amino-D-glucitol (Tables 2 and 3), revealed that (1→4)-linked glucose, (1→4) linked mannose, (→4)-GlcN-(1→). The linkages between the monosaccharides were predicted according to Bjorndal et al. [12, 27]. Based on the HPLC and GC-MS data, the probable structure of the ECP is given in Figs. 6 and 7.

**Table 2:** Results of the analysis of GC-MS

Fragments^a^	Mode of Linkage
1,2,4-tri-*O*-acetyl-3,6-di-*O*-methyl-D-mannitol	(→1, 2)-Man-(4→
1,4-di-*O*-acetyl-2,4,6-tri-*O*-methyl-D-glucitol	(→1)-Glc-(4→
1,4,6-di-*O-*acetyl-3-mono-*O*-methyl-2-amino-D-glucitol	(→1, 4)-GlcN-(6→

^a^Alditol acetates generated after methylation and reduction of monomers

**Table 3:** GC data showing the number of peak traces

Fragments	Mode of linkage	Major mass fragments (m/z) peak traces
1,2,4-tri-*O*-acetyl-3,6-di-*O*-methyl-D-mannitol	(→1, 2)-Man-(4→	104, 113, 129, 147, 161, 181, 191, 197
1,4-di-*O*-acetyl-2,4,6-tri-*O*-methyl-D-glucitol	(→1)-Glc-(4→	103, 119, 130, 151, 177, 193, 196
1,4,6-di-*O-*acetyl-3-mono-*O*-methyl-2-amino-D-glucitol	(→1, 4)-GlcN-(6→	110, 117, 131, 147, 163, 181, 191, 197

### Optimization studies of ECP production

#### Central composite design for medium optimization

Response surface methodology (RSM) is a collection of mathematical and statistical techniques widely used to determine the effects of several variables and to optimize different biotechnological processes [26]. The central composite design (CCD) was adopted for optimization of medium components such as glucose, yeast extract, and MgSO_4_. The experimental results of CCD for enhancing the yield of ECP are shown in Table 4.

**Table 4:** The nutritional variables selected for optimization study

S.No.	Nutritional variables	^a^Range in g/L
1	Glucose (A)	5–15
2	Yeast extract (B)	5–10
3	MgSO_4_ (C)	0.2–0.4

^a^Concentration ranges

The response (Y) fitted with the second-order polynomial equation

$$ {R}_1=+2.54+0.14\ A-0.032B-0.083\ C+0.014A\ B-0.13\;\mathrm{AC}-0.079\; BC-.59{A}^2-1.23\ {B}^2-0.11{C}^2 $$

**Fig. 8:**
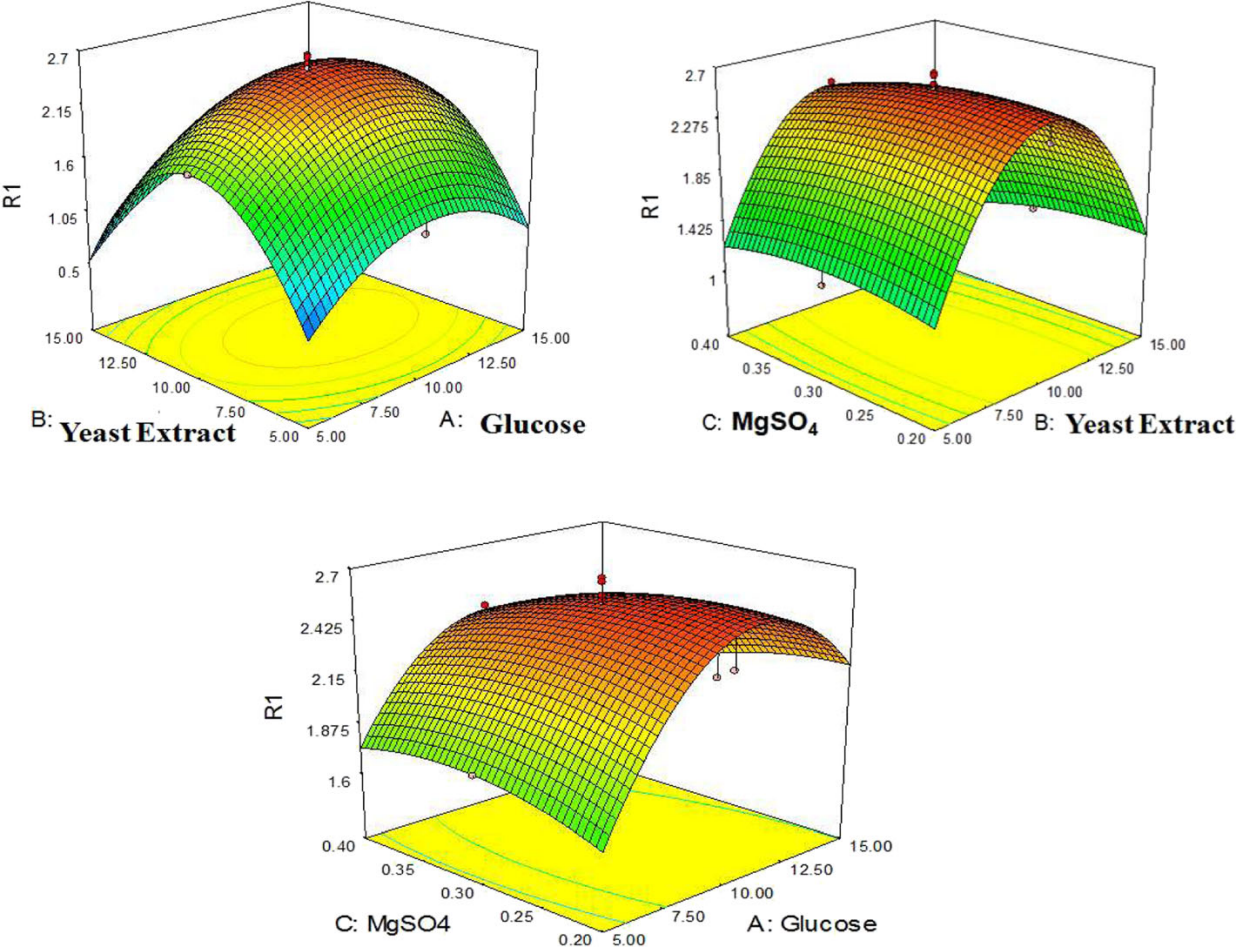
3D graphs showing the combinational interaction of variables on production of ECP

*R*_1_ represents the response for ECP production, whereas glucose, yeast extract, and MgSO_4_ are represented by variables *A*, *B*, and *C*, respectively, and the *R*^2^ coefficient value of 0.99 suggested that predicted model was significant (Table 5). The Model *F*-value of 154.37 implies the model is significant. In this case, *A*, *C*, AC, *A*^2^, and *B*^2^ are significant model terms (Table 6). The combinational effect of nutritional variables on the production of ECP was analyzed from the 3-D response surface curves as shown in Fig. 8.

**Table 5:** Actual data for design of experiments

S.No.	Glucose	Yeast extract	MgSO_**4**_	Production of biosurfactant in g/L	
				Experimental^a^	Predicted^b^
1.	15	10	0.3	1.95	2.1
2.	5	15	0.4	0.36	0.39
3.	10	10	0.3	2.51	2.54
4.	15	15	0.2	1.08	1.03
5.	5	5	0.4	0.63	0.64
6.	10	10	0.3	2.54	2.54
7.	10	10	0.4	2.36	2.34
8.	10	10	0.3	2.63	2.54
9.	10	5	0.3	1.22	1.34
10.	15	5	0.4	0.66	0.63
11.	10	15	0.3	1.25	1.28
12.	5	15	0.2	0.46	0.45
13.	10	10	0.3	2.65	2.54
14.	15	5	0.2	0.98	0.91
15.	10	10	0.3	2.56	2.54
16.	5	10	0.3	1.8	1.81
17.	10	10	0.3	2.65	2.54
18.	5	5	0.2	0.42	0.38
19.	15	15	0.4	0.44	0.44
20.	10	10	0.2	2.34	2.51

^a^Experimental result of biosurfactant production at the mentioned nutrient concentrations

^b^RSM predicted value of biosurfactant production at the mentioned nutrient concentrations

**Table 6:** ANOVA for response surface quadratic model

Source	Sum of squares	df	Mean square	F-value	***p***-value Prob > ***F***	
Model	15.38	9	1.71	154.37	< 0.0001	**Significant**^a^
A-glucose	0.21	1	0.21	18.73	0.0015	
B-yeast extract	0.01	1	0.01	0.92	0.3589	
C-MgSO_4_	0.07	1	0.07	6.22	0.0318	
AB	0.00	1	0.00	0.14	0.7194	
AC	0.14	1	0.14	12.92	0.0049	
BC	0.05	1	0.05	4.48	0.0604	
A^2^	0.95	1	0.95	85.92	< 0.0001	
B^2^	4.15	1	4.15	374.61	< 0.0001	
C^2^	0.04	1	0.04	3.18	0.1048	
Residual	0.11	10	0.01			
Lack of fit	0.09	5	0.02	4.95	0.0519	**Not significant**
Pure error	0.02	5	0.00			
Cor total	15.50	19				

^a^If *p*-value of a parameter is < 0.05, the effect of that parameter is significant

### Validation of RSM model

The RSM model is validated by conducting an experiment at best-predicted solution for production of ECP. Under optimized conditions, the ECP yield reached 2.21 g/L from *Acinetobacter indicus* M6, which is almost near to the RSM predicted value (Table 7).

**Table 7:** Validation of RSM model for ECP production

S.No.	Glucose (A), g/L	Yeast extract (B), g/L	MgSO_**4**_ (C), g/L	ECP production (R_**1**_), g/L	
				RSM model predicted	Experimental
1	15	10.12	0.2	2.19	2.21

## Discussion

*Acinetobacter* M6 produces ECP in substantial quantities (2.21 g/L). This quantity is considerably higher when compared to other reported bacterial ECPs. As far as our knowledge, there is no report available on ECP from *Acinetobacter indicus* M6 bacterium. ECP from this marine bacterium may be beneficial because few of the ECPs have been reported to have emulsification property which can find potential applications in the reduction of marine water pollution [27, 28]. The purified ECP shows significant antioxidant activities when compared to the standard antioxidant active compound (Vit C). Free radicals such as superoxide radical, hydroxyl radical, and other reactive oxygen species (ROS) are associated with multistage carcinogenesis and mutagenesis [29, 30]. The results of present study have demonstrated that ECP was effective in scavenging superoxide, hydroxyl, and DPPH radicals in a concentration-dependent fashion. There are very few reports available on antioxidant activities of bacterial ECPs. However, this is the first report on antioxidant and free radical scavenging activities of an ECP from *Acinetobacter indicus* M6*.* Hence, the ECP may be developed as a potential antioxidant molecule after studying various toxicological studies. The monosaccharides in the ECPs are actually potent reductive agents as they have a hidden aldehyde moiety [31]. The antioxidant mechanism of polysaccharides may thus be attributed to the reductive nature of the monosaccharides owing to the presence of –CHO group as these polysaccharides such as ECPs are not proton-donors. The mechanism of free-radical scavenging of polysaccharides is still not fully understood. The results of the present study have demonstrated that ECP is effective in scavenging superoxide, hydroxyl, and DPPH radicals in a concentration-dependent fashion. There are a very few reports available on antioxidant activities of bacterial ECPs. However, this is the first report on antioxidant and free radical scavenging activities of an ECP from *Acinetobacter indicus* M6. HPLC analysis indicated that ECP was a heteropolymer composed of glucose, mannose, and glucosamine. The presence of these monomers makes the heteropolymer unique, and this monomeric combination was not found in any other reported ECPs. GC-MS data of alditol derivatives revealed that (1→4)-linked glucose, (1→4)-linked mannose, and (→4)-GlcN-(1→) were present in the ECP. Presence of these linkages also makes our ECP unique and novel, and the predicted structure is given in Fig. 7 [32]. ECP from *Acinetobacter indicus* M6 was found to be a structurally complex but novel molecule for its unique monosaccharide composition and the glycosidic linkages between the monomeric units. This unique combination of the monosaccharides and unusual glycosidic linkages between them make our ECP novel. To undertake a holistic approach towards critically establishing the structure-function relationship of the ECP molecule by elucidating its complete molecular structure on further chemical derivatizations and enzymatic digestions (with glucosidases), so as enable us to identify the right fragments of the large ECP molecule, responsible for each of its important bioactive properties of therapeutic and commercial interests [27, 33]. This would also help us to use the small fragments for specific therapeutic purposes, instead of using the whole molecule [34, 35]. However, the complete structural elucidation may be performed by other sophisticated techniques like NMR and ESI-MS which is the scope of the present study. The optimized medium improved the production of ECP and is two folds higher in comparison with the basal medium.

## Conclusion

ECP produced by marine bacterium was purified and found to be a heteropolymer composed of glucose as a major monomer, and mannose and glucosamine were minor monomers, and this makes ECP unique in terms of its composition. GC-MS analyses elucidated the presence of quite uncommon (1→4)-linked glucose, (1→4)-linked mannose, and (→4)-GlcN-(1→) glycosidic linkages in the backbone. The purified ECP has shown significant antioxidant activity. The detailed mechanisms of actions of the ECP molecule for its significant antioxidant activities need to be elucidated to develop the whole ECP molecule or the suitable fragments of it into prospective drug candidates. This requires a lot of basic research inputs. The production of ECP reached 2.21 g/L after the optimization of nutritional variables. The designed model is statistically significant and is indicted by the *R*^2^ value of 0.99. The optimized medium improved the production of ECP and is two folds higher in comparison with the basal medium. As a long-term future scope and perspective, it would be prudent to scale the optimal process up to a pilot plant scale for consistent production of this important bioactive ECP molecule in large quantities. Though there are reports available on bacterial ECPs, this is the first report on ECP from *Acinetobacter indicus* M6. Unique monosaccharide composition and the glycosidic linkages between the monomeric units of the ECP markedly differ with the reported ECPs.

Considering the unique monosaccharide composition and significant antioxidant activity, the ECP may be developed as a drug molecule. The ECP is a promising antioxidant, and it can be used as an additive in food, pharmaceutical, and cosmetic preparations. The relationship between structure of ECP and antioxidant activity and also elucidation of its antioxidant mechanism at the molecular level will improve its biological activities by chemical modifications, one of the important implications of this study.

## Data Availability

The data is available and it will be provided to the editor if required.

## Abbreviations

ECP: Extracellular polysaccharide
DPPH: 1,1-diphenyl-2 picryl hydrazyl
RSM: Response surface methodology

## References

[1] Shabtai Y (1990). Production of exopolysaccharides by *Acinetobacter* strains in a controlled fed-batch fermentation process using soap stock oil (SSO) as carbon source. Int J Biol Macromol.

[2] Welman AD, Maddox IS (2003). Exopolysaccharides from lactic acid bacteria: perspectives and challenges. Trends Biotechnol.

[3] Naidu AS, Bidlack WR, Clemens RA (1999). Probiotic spectra of lactic acid bacteria (LAB). Crit Rev Food Sci Nutr.

[4] Ashtaputre AA, Shah AK (1995). Emulsifying property of a viscous exopolysaccharide from *Sphingomonas paucimobilis*. World J Microbiol Biotechnol.

[5] De Vuyst L, Bart D (1999). Heteropolysaccharides from lactic acid bacteria. FEMS Microbiol Rev.

[6] Kishk YFM, Al-Sayed HMA (2007). Free-radical scavenging and antioxidative activities of some polysaccharides in emulsions. LWT Food Sci Technol.

[7] Sun C, Wang JW, Fang L, Gao XD (2004). Free radical scavenging and antioxidant activities of EPS2, an exopolysaccharide produced by a marine filamentous fungus *Keissleriella sp.YS* 4108. Life Sci.

[8] Halliwell B, Gutteridge JMC (1986). Oxygen free radicals and iron in relation to biology and medicine: some problems and concepts. Arch Biochem Biophys.

[9] Reddy AR, Peele KA, Krupanidhi S, Kodali VP, Venkateswarulu TC (2018). Production of polyhydroxybutyrate from *Acinetobacter nosocomialis* RR20 strain using modified mineral salt medium: a statistical approach. Int J Environ Sci Technol.

[10] Wang QJ, Fang YZ (2004). Analysis of sugars in traditional Chinese drugs. J Chromatogr B.

[11] Donlan RM, Costerton JW (2002). Biofilms: survival mechanisms of clinically relevant microorganisms. Clin Microbiol Rev.

[12] Bjorndal H, Hellerqvist CG, Lindberg B, Svensson SA (1970). Gas-liquid chromatography and mass spectrometry in methylation analysis of polysaccharides. Chem Int.

[13] Gruter M, Leeflang BR, Kuiper J, Kamerling JP, Vliegenthart JF (1993). Structural characterisation of the exopolysaccharide produced by *Lactobacillus delbrückii* subspecies bulgaricus rr grown in skimmed milk. Carbohydr Res.

[14] Peele KA, Ch VRT, Kodali VP (2016). Emulsifying activity of a biosurfactant produced by a marine bacterium. 3Biotech.

[15] Dubois M, Gilles KA, Hamilton JK, Pebers PA, Smith F (1956). Calorimetric method for determination of sugars and related substances. Anal Chem.

[16] Wang H, Dong X, Zhou GC, Cai L, Yao WB (2008). In vitro and in vivo antioxidant activity of aqueous extract from *Choerospondias axillaris* fruit. Food Chem.

[17] Liu F, Ng TB (2000). Antioxidative and free radical scavenging activities of selected medicinal herbs. Life Sci.

[18] Ren D, Jiao Y, Yang X, Yuan L, Guo J, Zhao Y (2015). Antioxidant and antitumor effects of polysaccharides from the fungus *Pleurotus abalonus*. Chem Biol Interact.

[19] Nishimiki M, Rao NA, Yagi K (1972). The occurrence of superoxide anion in the reaction of reduced phenazine methosulfate and molecular oxygen. Biochem Biophys Res Commun.

[20] Sokmena M, Angelovab M, Krumovab E, Pashovab S (2005). In vitro antioxidant activity of polyphenol extracts with antiviral properties from Geranium sanguineum L. Life Sci.

[21] Anumula KR (1994). Quantitative determination of monosaccharides in glycoproteins by high-performance liquid chromatography with highly sensitive fluorescence detection. Anal Biochem.

[22] Anumula KR, Dhume ST (1998). High resolution and high sensitivity methods for oligosaccharide mapping and characterization by normal phase high performance liquid chromatography following derivatization with highly fluorescent anthranilic acid. Glycobiol.

[23] Kim SJ, Kim BG, Parka HU, Yim JH (2016). Cryoprotective properties and preliminary characterization of exopolysaccharide (P-Arcpo 15) produced by the Arctic bacterium *Pseudoalteromonas elyakovii* Arcpo 15. Prep Biochem Biotechnol.

[24] Maheswari P, Mahendran S, Sankaralingam S, Sivakumar N (2019) In vitro antioxidant activity of exopolysaccharide extracted from marine sediment soil bacteria. Res J Pharm Tech 12(9)

[25] Bomfim VB, Neto JHL, Leite KS, Vieira EA (2020). Partial characterization and antioxidant activity of exopolysaccharides produced by Lactobacillus plantarum CNPC003. Food Sci Tech.

[26] Lin SM, Baek CY, Jung JH, Kim WS, Song HY, Lee JH, Ji HJ, Zhi Y, Kang BS, Bahn YS, Seo HS, Lim S (2020) Antioxidant activities of an exopolysaccharide (DeinoPol) produced by the extreme radiation-resistant bacterium *Deinococcus radiodurans*. Nat Sci Rep 63(10):55 10.1038/s41598-019-56141-3 PMC6952347 31919371

[27] Yasuda T, Inaba A, Ohmori M, Endo T (2000). Urinary metabolites of gallic acid in rats and their radical scavenging effect on DPPH. J Nat Prod.

[28] Cheng BH, Chan JYW, Chan BCL (2014). Structural characterization and immunomodulatory effect of a polysaccharide HCP-2 from *Houttuynia cordata*. Carbohydr Polym.

[29] Venkateswarulu TC, Kodali VP, Kumar RB (2017). Optimization of nutritional components of medium by response surface methodology for enhanced production of lactase. 3 Biotech.

[30] Soares JR, Dins TCP, Cunha AP, Ameida LM (1997). Antioxidant activity of some extracts of *Thymus zygis*. Free Radic Res.

[31] Haschemie KK, Renger A, Steinhart H (1996). A comparison between reductive-cleavage and standard methylation analysis for determining structural features of galactomannans. Carbohydr Polym.

[32] Knight J (1998). Free radicals: their history and current status in aging and disease. Ann Clin Lab Sci.

[33] Sun T, Powers JR, Tang J (2007). Evaluation of the antioxidant activity of asparagus, broccoli and their juices. Food Chem.

[34] Kodali VP, Das S, Sen R (2009). An exopolysaccharide from a probiotic: biosynthesis dynamics, composition and emulsifying activity. Food Res Int.

[35] Venkateswarulu TC, Prabhakar KV, Kumar RB, Krupanidhi S (2017). Modeling and optimization of fermentation variables for enhanced production of lactase by isolated *Bacillus subtilis* strain VUVD001 using artificial neural networking and response surface methodology. 3 Biotech.

